# Restructuring the final year of the model study programme MaReCuM at the Medical Faculty Mannheim: the academic quarter in ambulatory medicine

**DOI:** 10.3205/zma001263

**Published:** 2019-10-15

**Authors:** Lisa Liebke, Elisabeth Narciß, U. Obertacke, Harald Fritz-Joas

**Affiliations:** 1 Medizinische Fakultät Mannheim der Universität Heidelberg, Geschäftsbereich Studium und Lehrentwicklung, Mannheim, Germany; 2 Medizinische Fakultät Mannheim der Universität Heidelberg, Mannheim, Germany

**Keywords:** model study programme, final year, ambulatory medicine, academic quarters, individual focus

## Abstract

**Introduction: **To reflect the ever-growing importance of outpatient care in medical education, MaReCuM – a reformed curriculum, also referred to as a model study programme – was introduced at the Medical Faculty Mannheim in 2006. It divided the final year of medical study into quarters and added a mandatory quarter dedicated to ambulatory medicine. This project report presents our experiences, the costs and the evaluation results connected with making specific changes to the final year of undergraduate medical study.

**Project description: **The final-year quarter in ambulatory medicine, taught at the Medical Faculty’s outpatient teaching placements, allows final-year medical students to gather practical experience in one of four elective areas in outpatient care. The parallel coursework encompasses interactive case presentations and practical reviews. Relevant curricular content on ambulatory medicine is then tested in the oral/practical section of the M3 medical examination.

**Results:** Students are very satisfied with the academic quality of the final-year quarter in ambulatory medicine. Restructuring the final year, generating the concept and recruiting teaching placements at outpatient facilities required additional full-time positions in the beginning.

**Discussion: **The processes of reforming MaReCuM have not only contributed to a stronger recognition of ambulatory medicine in the final year and in the clinical phase of study, but have also enabled broader opportunities for focussing individual choices during medical education. A high caliber of academics in the quarter in ambulatory medicine can be achieved with a calculable amount of organisational effort.

**Conclusion: **Anchoring a curriculum on ambulatory medicine in medical education is possible through restructuring the final year and is received positively by students. The success of MaReCuM demonstrates the feasibility of the recommendations made by the German Council of Science and Humanities (Wissenschaftsrat).

## Zusammenfassung

**Einleitung:** Um den steigenden Stellenwert der ambulanten Patientenversorgung in der medizinischen Ausbildung abzubilden, wurde an der Medizinischen Fakultät Mannheim im Jahr 2006 ein Modellstudiengang eingeführt, dessen Kern in der Quartalisierung des Praktischen Jahres (PJ) und der Einführung eines Wahlpflichtquartals Ambulante Medizin besteht. Unser Projektbericht stellt die Erfahrungen sowie die Kosten und die Evaluationsergebnisse der Umsetzung der Änderungen im Praktischen Jahr dar.

**Projektbeschreibung: **Das PJ-Quartal Ambulante Medizin in ambulanten Lehreinrichtungen der Fakultät vermittelt PJ-Studierenden Praxiserfahrung in einem von vier wählbaren ambulanten Bereichen. Der begleitende Unterricht umfasst interaktive Fall-Referate und Praxis-Repetitorien. Die Inhalte des Quartals Ambulante Medizin werden im mündlich-praktischen Teil des M3-Examens geprüft.

**Ergebnisse: **Die Studierenden sind mit der Qualität der Ausbildung im PJ-Quartal Ambulante Medizin sehr zufrieden. Die Umstrukturierung des PJ, die Erstellung des Konzepts und die Akquise der ambulanten Lehrplätze erforderte zumindest initial zusätzliche Vollkostenstellen.

**Diskussion:** Die Reformprozesse im Modellstudiengang haben sowohl zu einer verstärkten Anerkennung der ambulanten Medizin im Praktischen Jahr und im klinischen Studium als auch zu einer erweiterten Neigungsorientierung im Studium beigetragen. Eine hohe Ausbildungsqualität im Quartal Ambulante Medizin kann mit einem kalkulierbaren organisatorischen Aufwand erreicht werden.

**Schlussfolgerung:** Eine Verankerung ambulanter Inhalte im Medizinstudium ist durch eine Umgestaltung des PJ möglich und wird von den Studierenden positiv bewertet. Die erfolgreiche Anwendung des Modellstudiengangs belegt die Machbarkeit der Empfehlungen des Wissenschaftsrats.

## 1. Introduction

### 1.1. The concept behind and approval of Medical Faculty Mannheim’s model study programme

The model study programme – MaReCuM – was implemented at the Medical Faculty Mannheim in the fall of 2006. Prior to this, in 2003, an expert statement was issued by the German Council of Science and Humanities (Wissenschaftsrat) approving the expansion of the Faculty of Clinical Medicine in the areas of basic research and teaching. In December 2005 the Senate and the Faculty Council passed a resolution to expand the Faculty of Clinical Medicine to a comprehensive medical faculty covering both preclinical and clinical training. Since Heidelberg University at that time effectively had two medical faculties, this opportunity was also used to implement a model study programme in Mannheim – MaReCuM – alongside Heidelberg’s reformed medical curriculum, HeiCuMed. The establishment of model study programmes is provided in Section 41 of the German Medical Licensing Regulations (ÄAppO).

The concept behind the study programme mainly envisioned elevating the importance of ambulatory medicine in medical education. The petition for approval as a model study programme is based on Section 41(3) of the ÄAppO in the form of a modification to the curriculum in the final year of medical study. The new concept is a final year that is divided into four quarters (12-week rotations) in order to introduce a new, mandatory quarter dedicated to ambulatory medicine with elective areas (petition submitted by Heidelberg University on 11 April 2006) and thus also strengthen the elective options during the final year [see [1], [2]]. To improve the academic quality of the final year and develop a reformed final-year curriculum, a Competency Centre to improve final-year academics at the medical faculties in Baden-Wuerttemberg was founded on 13 July 2005 in Mannheim with the support of the state government. The following contains a brief overview of the model curriculum and then outlines the specifics of dividing the final year into quarters. The special features of the quarter in ambulatory medicine and the possibility to set an individual focus, as well as the costs and benefits, are likewise presented and discussed.

#### 1.2. Overview of the most important characteristics of MaReCuM

**Figure 1:**
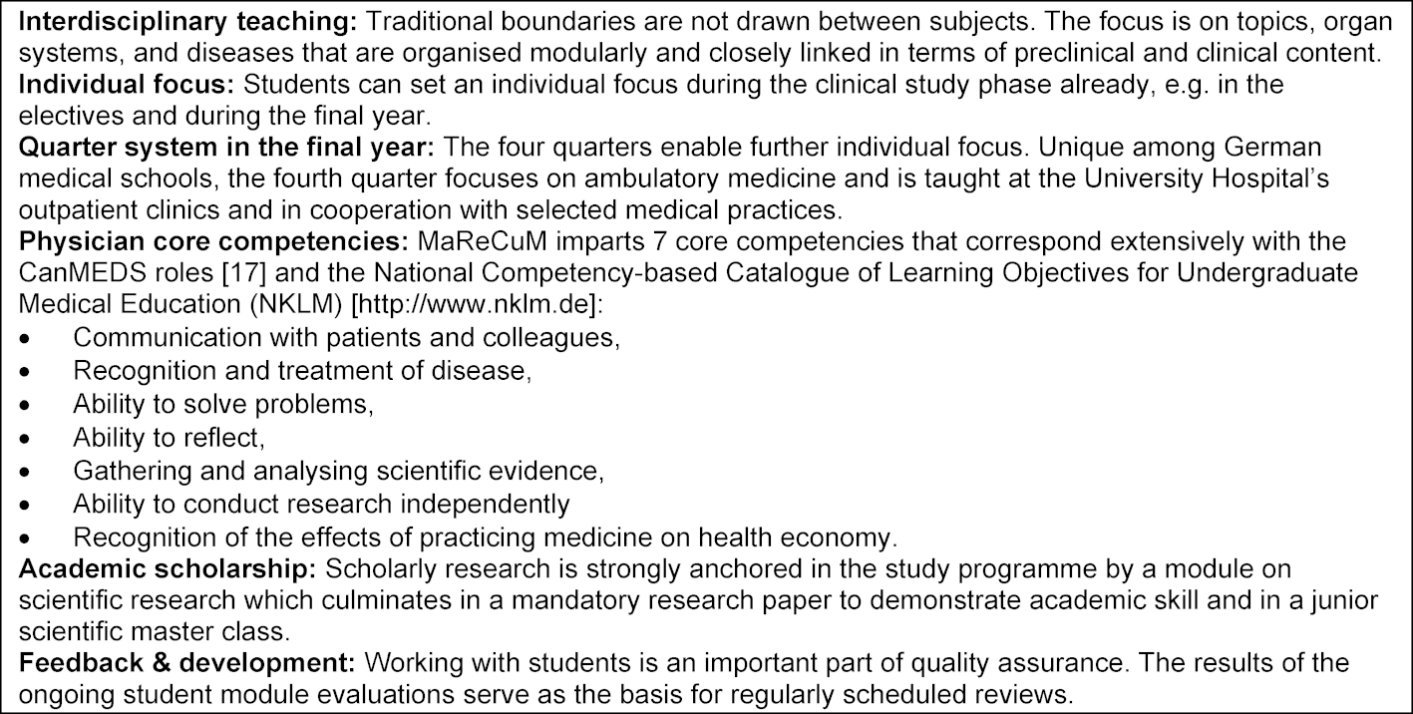
Characteristics of the model study programme MaReCuM.

The introduction of preclinical studies at the Medical Faculty Mannheim, which occurred at the same time as the implementation of the model curriculum, led to a uniform concept across the entire programme. The Centre for Biomedicine and Medical Technology (CBTM) was created and blurred traditional subject boundaries by foregoing separate institutes and embracing modular, topic-specific structures in the curriculum and at the institutional level. In addition, a vertical networking of preclinical and clinical content was ensured from the very start: each preclinical module was assigned a clinical mentor, for instance a trauma surgeon in the topic block covering the musculoskeletal system, a gastroenterologist in the block covering the digestive tract, etc. In the initial planning stages, the Medical Faculty had already identified the principles that the model study programme should embody and defined core competencies with which the curricular content should align. The characteristics of MaReCuM are presented in figure 1 (Fig. 1).

With the restructuring of the final year, the medical curriculum in Mannheim now differs from all other reformed, or model study programmes in Germany. This key difference is that students no longer need to sit for the first part of the State Medical Examination. The reason for retaining the M1 medical examination in Mannheim was primarily to ensure comparability with conventional study programmes, since national benchmarking was of special interest for the Medical Faculty Mannheim in light of its innovative preclinical study phase. Moreover, the ability of students to transfer from one medical school to another needed to be maintained. After implementing MaReCuM in the fall of 2006, the first cohort of the new study programme took the M1 medical examination in 2008. In the national comparison of test scores among the medical schools, MaReCuM always placed within the top four during the first ten fall semester examinations.

#### 1.3. MaReCuM’s final year: ambulatory medicine, academic quarters and individual focus

Medical care is increasingly shifting from the hospital setting to the outpatient sector [3]. This development was encouraged in 1992 already by German legislation affecting the structure of healthcare; it has been pushed further since then by additional legislation and all key actors (service providers and recipients, including patients) [4]. An increasing number of patients with acute, life-threatening diseases (transplantation, oncology, HIV, etc.) survive and experience long and chronic disease progression as outpatients (dialysis, chemotherapy, etc.). Medical and technological progress [5] has enabled diagnostics and therapy to take place increasingly in outpatient clinics and medical practices, something that is reflected at Mannheim’s University Hospital: in 2017, 51,678 inpatients and 215,197 outpatients were treated [6].

Efforts to stay abreast of this very real change in healthcare and to faithfully reflect it in medical education are vital [7], [8], [9], [10]: an insufficient representation of ambulatory medicine in all of the clinical subjects taught at medical school, while an even more massive shift of inpatient care to the outpatient sector takes place, runs the risk that students will no longer come into contact with important clinical pictures, courses of disease or therapeutic processes during their medical studies [8].

In Mannheim these developments were met with a modification of the final year. These changes (dividing the final year into quarters, dedicating a quarter to ambulatory medicine, greater opportunity to choose an individual focus), which have been in place in Mannheim since 2011, are now being explicitly called for in the Master Plan 2020 for undergraduate medical education that was issued in March 2017 by coalitions and the German Federal Ministry of Health [11]. This plan states that since the provision of healthcare is increasingly shifting from hospitals to the outpatient setting, we must also incorporate this development into medical education. Physicians-to-be must, as a result, become familiar not only with the highly specialized cases seen at university hospitals and which have been the central focus of medical training to date, but also with the diseases routinely treated in both the inpatient and outpatient settings [11]. The German Council of Science and Humanities also views a stronger inclusion of outpatients in teaching as necessary and explicitly recommended, in 2014 already, the division of the final year into quarters and encouraging individual focus in the form of a fourth elective in the curriculum [12].

Confronted with the quarter system envisioned by the Master Plan 2020 and the formal inclusion of ambulatory medicine, other medical faculties are now faced with a similar restructuring of the final year and could profit from our experiences. Concerns still exist that imposing a quarter system on the final year could increase organisational efforts and compromise academic quality (see the concerns expressed by the German Medical Association [Bundesärztekammer] [13] and other professional medical associations [14]).

#### 1.4. Issues regarding the changes to the final year

- How can imposing a quarter system on the final year and the implementation of a new quarter be realized? (See sections 2.1 and 2.2 for a detailed project description, including teaching positions with a list of criteria for delineating the separate quarters and examples of important clinical pictures treated in the outpatient setting, etc.)
- How does restructuring the final year contribute to placing greater importance on ambulatory medicine in the medical curriculum? (See section 2.2 for a description of the outpatient teaching placements and the accompanying coursework; section 2.3 for how ambulatory medicine is anchored in the M3 medical examination; and sections 2.4 and 3.1 for the results of the student evaluations regarding their willingness to take this mandatory quarter.)
- Are the students satisfied with the academic programme in the final-year quarter in ambulatory medicine? (See sections 2.4 and 3.1 for the results of the student evaluations regarding their satisfaction with the final-year academic programme in the different quarters.)
- Does the quarter system in the final year enable students to pursue an individual focus more in depth? (See section 2.2 for a description of the elective areas and sections 2.4 and 3.1 for the results of the student evaluations regarding their assignment to the elective areas.)
- How high are the effort and cost associated with implementing the quarter system in the final year and the introduction of a mandatory quarter in ambulatory medicine? (See sections 2.5 and 3.2 for a calculation of the costs of implementing, coordinating and organising the new final-year quarter.)

## 2. Project description: implementation of key measures in the final year of MaReCuM

After the initial start of MaReCuM in the 2006/07 winter semester, the first cohort entered its final year and, as a result, the quarter in ambulatory medicine in August 2011. This allowed the Medical Faculty and the Competency Centre time to prepare for the restructuring of the final year and plan comprehensive quality management.

### 2.1. The final year in academic quarters

Redesigning the final year meant replacing the existing trimesters of 16 weeks each with four quarters of 12 weeks each.

“Synchronizing” the final-year quarters was particularly helpful in terms of planning schedules and organising teaching: the first (second) quarter of the current cohort and the third (fourth) quarter of the previous cohort begin at the same time. This synchronization allows for two scheduled breaks of one week that are inserted for the Christmas and Easter breaks, so that while the final year is 50 weeks long, it only has 48 weeks of attendance. Also, the State Examination Office (Landesprüfungsamt or LPA) has stated that a quarter must be 10 weeks minimum, with consequences for absences and splitting quarters. As before, completing part of the final year abroad is possible; however, LPA requirements state that up to two quarters may be completed as study abroad. Of these, it is possible to split one quarter into eight weeks of study abroad and four weeks of attendance at the University Hospital Mannheim.

#### 2.2. The final-year quarter in ambulatory medicine

In the new mandatory quarter in ambulatory medicine, students have the opportunity to familiarize themselves with the ongoing diagnostics and therapies provided in outpatient healthcare through repeated contact with patients presenting at outpatient clinics and medical practices. It is in this context that medical students can see clinical pictures that they have not yet seen in the hospital setting and learn about special aspects of providing outpatient care, for instance, discerning targeted diagnostic measures and therapies in a limited amount of time for a previously unknown patient or planning long intervals between check-ups [15]. In this way, the final-year quarter in ambulatory medicine draws and builds on prior experiences with outpatients, for instance, the block practicum in general practice taken during the fifth year of study. These later study phases lend themselves especially for integrating ambulatory medicine because it is during these phases that students’ clinical knowledge and practical skills are sufficiently developed enough to be applied to real patients.

Currently, as of March 2019, the final-year quarter on ambulatory medicine can be served at a total of 64 teaching placements housed in the University Hospital’s outpatient clinics and other outpatient clinics and medical practices (see table 1 (Tab. 1)). To allow students to pursue professional pathways in selected areas within ambulatory medicine, it is possible to choose between four areas that, in turn, cover diverse subject areas (no change in area over the 12 weeks):

- Surgical/interventional medicine
- Conservative/chronic medicine
- Oncology
- Psychiatry/psychotherapy

The final-year quarter in ambulatory medicine covers specialized outpatient care in these areas. Aspects of primary care in the traditional primary care disciplines such as internal medicine, pediatrics, general practice, gynecology, (trauma) surgery, ENT medicine, etc. have been taken into particular account. For instance, the teaching settings for internal medicine address cardiology, nephrology and diabetology, in which clinical pictures relevant to primary care are treated (coronary heart disease, chronic liver disease, diabetes, etc.). The aim is to expand the number of primary care subjects covered.

Each of the teaching placements is described with a brief summary of the patient clientele and the curricular content that can be learned. In addition, each site is evaluated to verify that specific learning criteria for the final-year curriculum are met. The latter represents further development of the criteria for general practice in the final year and was prepared by the Medical Faculty in close collaboration with LPA as the supervisory body. Special attention was paid not only to the curricular content of the placements, but also to the academic qualifications and connections of the teachers to the Medical Faculty (meaning a large part of the teaching placements fall under the responsibility of the University Hospital’s outpatient clinics and/or physicians who serve as professors at the Medical Faculty Mannheim; see table 1 (Tab. 1)).

There is usually a student-teacher ratio of 1:1 during the practical training in the final year, since a clear assignment of tasks for the mentor teachers is necessary to meet the teaching placements’ descriptions and the requirements of the ambulatory medicine logbook. This means that in the vast majority of cases only one student can be trained at a placement at one time. Mandatory conferences at the beginning and end of the quarter and a minimum of three structured observations of the student with feedback are required during the student’s tenure at the teaching placement.

“Ambulatory Wednesdays”, specifically tailored to the outpatient setting, have been introduced as a new teaching format to accompany the quarter in ambulatory medicine in the form of a longitudinal curriculum and are held on the campus of Mannheim’s University Hospital. These entail 10 half-days during the quarter on which the students present interactive case reports on cases they have seen in outpatient care after which they field questions by previously appointed debate partners or a panel. The mentors at each site where the ambulatory quarter is taught supervise the presentations and discussions. In addition, training sessions on practical skills take place as practical reviews in conjunction with the Ambulatory Wednesdays (e.g. basic life support, electrocardiography, hygiene/suturing, etc.).

#### 2.3. Anchoring ambulatory medicine in the M3 examinations

In compliance with the stipulations laid down by the administrative body governing the model study programme, one of the four examiners for the oral/practical section of the M3 examination is a representative of ambulatory medicine and ensures that the issues pertaining to ambulatory medicine are adequately addressed in the examination, if needed in cooperation with the head of the examination committee. This may take place within the scope of the examiner’s own area of specialty or within the context of questions that are interdisciplinary or about healthcare issues; for instance, the examinees must identify which diagnostic and therapeutic measures could apply to certain outpatients or how the continuity and quality of treatment of a patient can be ensured in the ambulatory setting after hospital discharge.

#### 2.4. Student evaluations of the final year

The Office of the Dean of Studies administers student evaluations at the end of each quarter of the final year. The Mannheim questionnaire on satisfaction with experiences in the final year (Ma-FEZ-PJ) [16] uses 27 items and a five-point Likert scale to assess study conditions and satisfaction with a particular final-year quarter. The mean values for this questionnaire were analysed for the final-year cohorts from 2012 to 2018 (n=2073 quarters). High values represent a high level of quality or satisfaction with the final year at the teaching placement from the student perspective. Differences in the evaluation results between the four subjects of the final-year quarters were determined using repeated measures analysis of variance and post hoc pairwise comparisons with Bonferroni correction.

Furthermore, these students responded to additional questions as part of the evaluations conducted since the 2014/15 winter semester. To ascertain if students desire more in-depth training in ambulatory medicine and what importance they place on it, students were asked if they would have chosen to take the final-year quarter in ambulatory medicine if it were not mandatory. The question whether students were assigned to their desired areas within the quarter in ambulatory medicine was meant to provide information about the extent to which individual focus was being realized. Both questions were formulated as yes/no questions; the number of affirmative answers was tallied.

#### 2.5. Costs of the restructuring

To estimate the costs of restructuring the final-year curriculum to include the quarter in ambulatory medicine and offering it on an ongoing basis, the funds for generating the concept, implementation, coordination and organisation were quantified. When calculating this, it is important to take into account the relevant numbers of enrolled students. To prepare for the switch to quarters and the introduction of a new quarter, the initial acquisition and accreditation of 45 outpatient teaching placements for 180 final-year students each year was undertaken in parallel with conceptualization. This one-time effort that spanned two and a half years is considered separately from the long-term and ongoing organisation necessary for the Ambulatory Wednesdays and the assignment of students to their desired areas. An additional 19 teaching placements have been steadily recruited since then to give students more elective choice and to accommodate what are now 240 students per year.

## 3. Results

The Medical Faculty Mannheim looks back on 12 years of experience with – and data on – the MaReCuM curriculum. At the end of October 2018, a total of 1,070 students, in 13 cohorts, have passed through the final year under the quarter system with the quarter in ambulatory medicine.

The organisational changes described here and the conditions imposed by LPA were put into practice without complications.

### 3.1. Student evaluations of the final year

**Figure 2:**
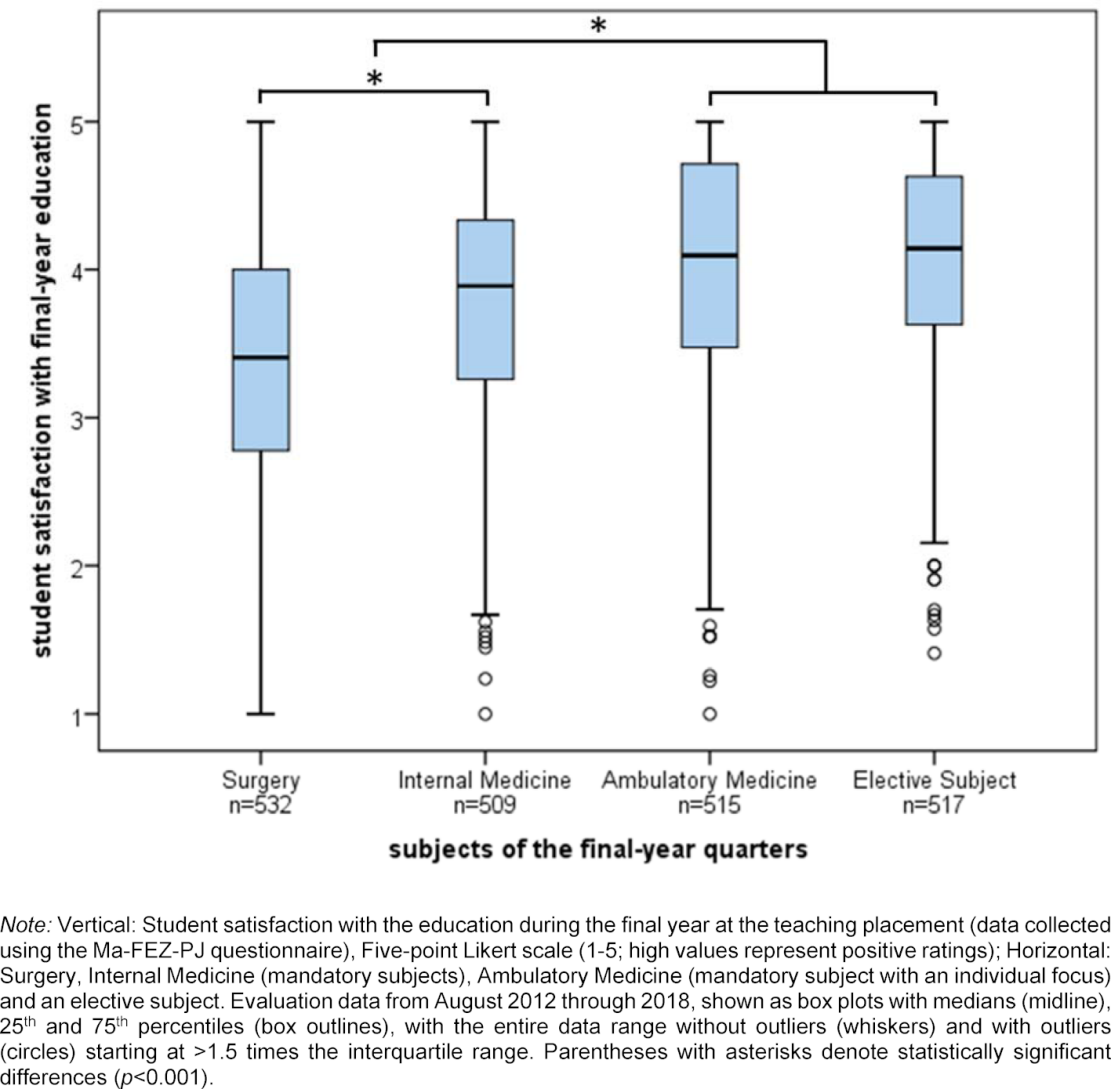
Student evaluations of the education in the individual final-year quarters.

Student satisfaction with the final-year curriculum differed between the subjects, F(3, 2069)=69.9, p<0.001. The quarter in ambulatory medicine was rated as positively as the elective subject (p=0.722); both of these quarters were rated more positively than the two other mandatory quarters (surgery, internal medicine) (all p<0.001). The distribution of the evaluations is illustrated in figure 2 (Fig. 2).

As of the 2014/15 winter semester, 61% of a total of 337 students reported that they would choose to take the quarter in ambulatory medicine even if it were not mandatory. In regard to the individual focus, 92% of 348 students during this time period indicated that they were assigned to the area of their choice within the quarter in ambulatory medicine.

#### 3.2. Cost of implementation

After being accepted by the Medical Faculty as a teaching placement and fulfilling the criteria for accreditation, medical practices receive formal recognition as academic outpatient clinics; this special recognition can be used in business communications and on certificates. Financial compensation does not exist. Professors on the medical faculty who teach at accredited medical practices (n=13) receive two semester credits toward their required teaching commitments if they can demonstrate having mentored two final-year students per semester.

One full-time academic position was required for at least two and a half years to fulfil the organisational tasks at the Medical Faculty Mannheim to prepare for implementation; afterward in the course of ongoing administration this became a half-time position dedicated to further (quality) development, the recruitment of new teaching placements, and administration (10% of time), plus a student employee working 20 hours per month.

## 4. Discussion

Experience with implementing the restructured MaReCuM curriculum demonstrates that it is possible to introduce a quarter system for the final year that also enables the integration of ambulatory medicine into the mandatory curriculum during the final year. Individual focus within ambulatory medicine is possible through a choice of different areas in outpatient care. Moreover, the teaching of ambulatory medicine can take place at the University Hospital’s outpatient clinics and the medical practices of faculty members such that the outpatient teaching institutions are included in the academic endeavors as required by LPA. Imposing the quarter system and introducing the quarter dedicated to ambulatory medicine were possible without additional costs, except for the extension of staff positions for concept and quality development and organisation. From the evaluation results presented here it is clear that the students are very satisfied with the academic quality of the new quarter in ambulatory medicine, even as satisfied as with the elective subject they have chosen.

### 4.1. Dividing the final year into academic quarters: organisational effort and educational quality

The concerns regarding the implementation of a quarter system that have been expressed from various positions [14], [15] can in our experience be viewed as follows. Regarding organisational aspects and tasks, the calculation of costs indicates that additional staff positions are indeed necessary to realize the changes to the final year. Optimistically, after successful implementation of the concept, the ongoing administration of the mandatory final-year quarter can be done as a minimal part of the position’s responsibilities and also without any additional costs, for instance, in connection with training at the outpatient clinics or medical teaching practices. Synchronizing the final-year quarters significantly reduces organisational effort. The evaluation results (and examination scores) show that the educational quality of the final year has not decreased. It must be noted, however, that this predominantly reflects student perspectives. Systematic validation by an external body to show that the quality criteria for teaching in the final year are being met would be desirable in the future. It is also suspected that the equal ratio of teachers to students in the quarter in ambulatory medicine specifically adds to the students’ level of satisfaction. It would be interesting to investigate how a smaller student-teacher ratio in other subjects could affect the (subjectively perceived) quality of education.

#### 4.2. The new final-year quarter: the significance of ambulatory medicine and individual focus

Implementing MaReCuM has successfully anchored the increasing importance of ambulatory medicine in the medical curriculum of the Medical Faculty Mannheim. This development is indicative of the future since the outpacing of the hospital sector by the outpatient sector will continue to progress even further [5]. It can be assumed that the students studying medicine at the Medical Faculty Mannheim are aware of the significance of ambulatory medicine (all specialties). This is indicated by evaluation results showing that over half of the students would choose to take the final-year quarter in ambulatory medicine if it were not already mandatory. That a vast majority of the students report that they were assigned to the area of their choice while taking the quarter in ambulatory medicine makes it very clear that it is indeed possible to create individual pathways in the clinical study phase. While switching to a quarter system did entail shortening the length of the elective by four weeks, the additional elective areas associated with the quarter in ambulatory medicine nonetheless strengthen the individual focus. For instance, general practice can also take on greater importance during the final year as an elective subject in one quarter of the final year and in addition as a focus within the quarter in ambulatory medicine, e.g. conservative/chronic medicine.

To further develop the model study programme, even stronger integration of ambulatory medicine prior to the final year would be desirable. For this reason, the Medical Faculty Mannheim is presently putting forth effort to link elements of ambulatory medicine to the curriculum in the early phase of clinical study (longitudinal module on ambulatory medicine). Funding for this has been received from the German state of Baden-Wuerttemberg. However, it continues to remain unclear if the stronger emphasis on ambulatory medicine in the medical curriculum ultimately leads to more students who later seek professional careers in the outpatient sector.

#### 4.3. Impediments and opportunities in the reform process

To implement the reforms described here, the creation of a Baden-Wuerttemberg Competency Centre, specifically dedicated to the final year of medical study, and the university-led Working Group on Curricular Reform have been crucial. Of advantage for the entire reform effort was certainly the small size of the school with only 150 students in 2000 when it was at risk of being left behind at the state level because the German Council of Science and Humanities had identified deficiencies in basic research. The precariousness of its situation turned out to be a strongly motivating force behind the desire to establish a particularly innovative model study programme, to some extent as a justification for the school’s existence. The developments that took place over the course of implementing the model study programme contributed and still contribute to the fact that the Medical Faculty Mannheim recognizes the value of good teaching.

## 5. Conclusion

This project report allows an estimation of the feasibility of imposing a quarter system on the final year of undergraduate medical study and the anchoring of ambulatory medicine (of diverse subjects) and individual focus while sharing important points related to the concrete realisation of such measures. However, not all of the questions raised in connection with this can be answered empirically, indicating that further studies would be desirable.

The final-year quarter in ambulatory medicine achieves the important integration of ambulatory teaching placements and patients into the education and training of future physicians. The intensification of individual focus within the medical curriculum is very possible at minimal cost. In particular, the high level of satisfaction among students with the education offered during the final-year quarter in ambulatory medicine optimistically corresponds to the acceptance of the greater importance placed on ambulatory medicine by the students. The major changes applied to MaReCuM (quarter system for the final year and a stronger anchoring of ambulatory medicine), as approved of by the model study programme clause contained in the ÄAppO, are now found in nationwide recommendations and the initial groundwork for fundamentally amending and revising the ÄAppO [11].

## Acknowledgements

As Dean of Studies, Prof. Dr. Harald Klüter oversaw the implementation of MaReCuM. We also wish to thank Dr. Katrin Schüttpelz-Brauns for critically reading the paper.

## Competing interests

The authors declare that they have no competing interests.

## Figures and Tables

**Table 1:**
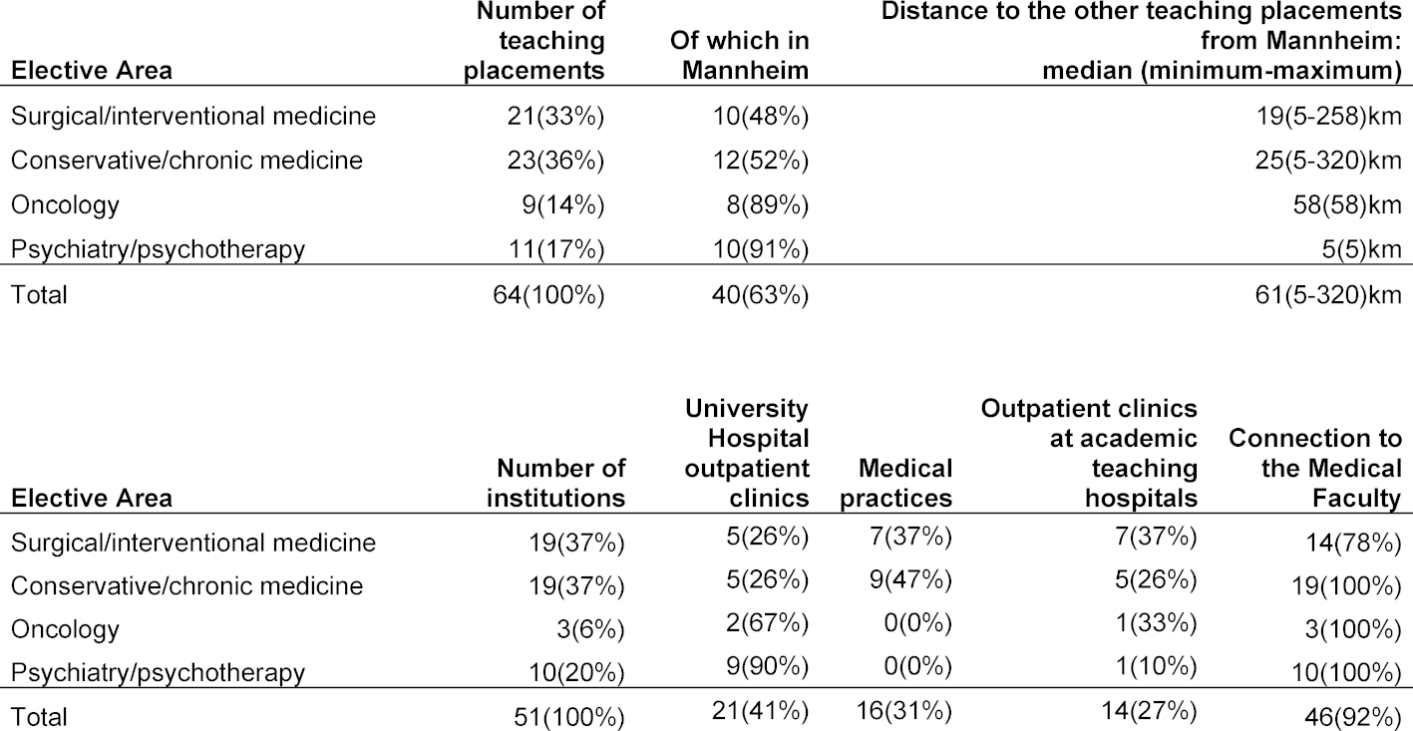
Overview of the outpatient teaching placements (rounding error of 1% maximum)
